# Shrinking follow-up duration in pilonidal sinus surgery, 1970–2020: a patient-weighted longitudinal analysis of 2,285 studies

**DOI:** 10.1007/s00423-026-04027-0

**Published:** 2026-03-29

**Authors:** N Cigdem Arslan, Jannik Seifert, Dietrich Doll

**Affiliations:** 1 Department of Surgery, Istanbul Health and Technology University, Hakki Yeten cad. No:13/68, Sisli, Istanbul, 34394 Turkey; 2 Vechtaer Institut für Forschungsförderung VIFF e.V., Vechta, Germany; 3 Department of Procto-Surgery & Pilonidal Sinus, St. Marienhospital Vechta, Academic Teaching Hospital of the MHH Hannover, Vechta, Germany; 4 Pilonidal Network for Expertise, Research and Development Collaboration Group, Vechta, Germany

**Keywords:** Pilonidal sinus disease, Follow-up duration, Recurrence, Longitudinal trend, Core outcome set

## Abstract

**Background:**

Reliable estimation of recurrence after pilonidal sinus surgery requires sufficient follow-up (FUP). Despite evidence suggesting ≥ 5 years is necessary, contemporary reports appear to adopt progressively shorter surveillance windows.

**Methods:**

We assembled a structured database of 2,285 pilonidal surgery series (1850–present) and extracted publication mid-decade, FUP since surgery, and cohort size (n). For 1970–2020, we calculated decade-wise patient-weighted mean FUP. Secular change was estimated using weighted least squares (WLS; weights = n), with unweighted sensitivity analyses. Associations between decade and FUP were assessed via weighted Pearson correlation with Kish effective sample size. We summarized proportions meeting ≥ 2, ≥5, ≥ 10 years and patient shares by FUP bands.

**Results:**

Median FUP was 1.67 years; mean 2.50 years. Only 14.2% of studies reported ≥ 5 years and 2.9% ≥10 years. Patient-weighted mean FUP peaked in the 1980s at 6.69 years, declining to 5.26 (1990s), 4.26 (2000s), 3.14 (2010s), and 2.55 years (2020s). Relative to the 1970s (3.78 years), the 2020s were − 1.23 years (− 32.5%), and − 4.14 years (− 61.9%) below the 1980s. The WLS slope was − 0.0766 years·year⁻¹ (95% CI − 0.0856 to − 0.0675; *p* = 4.57 × 10⁻⁵⁸); unweighted slope − 0.0407 (95% CI − 0.0479 to − 0.0334; *p* = 1.47 × 10⁻²⁷). Weighted correlation: Rw = − 0.342 (Kish *n* ≈ 216; *p* = 2.5 × 10⁻⁷). In the 2020s, patients were distributed as 55.3% <2 years, 26.6% 2–<5, 15.8% 5–<10, 2.3% ≥10.

**Conclusions:**

Follow-up windows have contracted substantially, risking under-ascertainment of late recurrences. A minimum of ≥ 5-year FUP with staged reporting at 5 and 10 years and registry-based surveillance is recommended. The absence of a pilonidal core outcome set (COS) in guidelines impedes consistent, long-horizon reporting; COS development and guideline adoption should be prioritized.

## Background

Pilonidal sinus disease (PSD) affects predominantly young adults, imposing a chronic burden through pain, time off work and frequent reoperations. Despite a century of evolving operative strategies—from radical excision with open healing to modern so called minimally invasive endoscopic techniques—recurrence remains a clinical nemesis. The assessment of any technique’s durability hinges critically on the length of postoperative surveillance [1]. Multiple prospective cohorts and meta‑analyses have demonstrated that early recurrence‑free rates (< 24 months) are unreliable proxies for ultimate success, with late failures surfacing up to a decade after wound closure [1–3]. Consequently, recent guideline drafts have advocated a minimum follow‑up (FUP) duration of five years and strongly encourage ten‑year data whenever feasible [4, 5].

Paradoxically, anecdotal evidence suggests that modern PSD studies reflected in the published literature are trending toward shorter observation windows. Two structural gaps contribute to this problem. First, there is no PSD core outcome set (COS) endorsed by guidelines that standardize outcome definitions, ascertainment methods, and minimal FUP time points. In contrast to other surgical domains, the absence of a COS for PSD fosters heterogeneity in recurrence definitions, timing, and reporting completeness, limiting comparability and meta-analytic synthesis. Second, journal and funding pressures incentivize shorter timelines, which can bias perceived effectiveness toward techniques with delayed failures.

We therefore conducted a longitudinal, patient-weighted analysis. We hypothesized that FUP durations have progressively contracted, and that the proportion of evidence meeting long term has fallen to levels that threaten accurate estimation of late recurrence. We further outline methodological recommendations—including COS development and registry-based surveillance—to restore decision-grade reporting.

## Methods

### Study design and protocol

We conducted a longitudinal bibliometric–epidemiologic review of 2,285 pilonidal surgery series to quantify secular changes in FUP length over five decades (1970–2020) to quantify temporal trends in reported FUP duration after pilonidal sinus surgery. The study was designed a priori in accordance with PRISMA 2020 principles for transparent reporting. The protocol specified objectives, eligibility criteria, data items, and statistical analyses, and was finalized before screening commenced. As this study synthesized published, study-level data and did not involve identifiable human participants, institutional ethics approval was not required.

### Data sources and search strategy

We systematically searched MEDLINE (via PubMed), Embase, Scopus, Web of Science Core Collection, and Cochrane CENTRAL from inception to the search date, without initial language or date restrictions. We complemented database searches with Google Scholar screening for grey literature and citation chasing, as well as manual review of reference lists from key reviews and eligible studies. The PubMed strategy combined terms for pilonidal disease and surgical management with outcome and FUP terminology (e.g., “pilonidal” OR “pilonidal sinus” AND “surgery” OR “excision” OR “flap” OR “endoscopic” AND “FUP” OR “recurrence” OR “outcomes”) and was adapted to controlled vocabularies in each database. All records were exported and de-duplicated prior to screening.

### Eligibility criteria and study selection

**Fig. 1:**
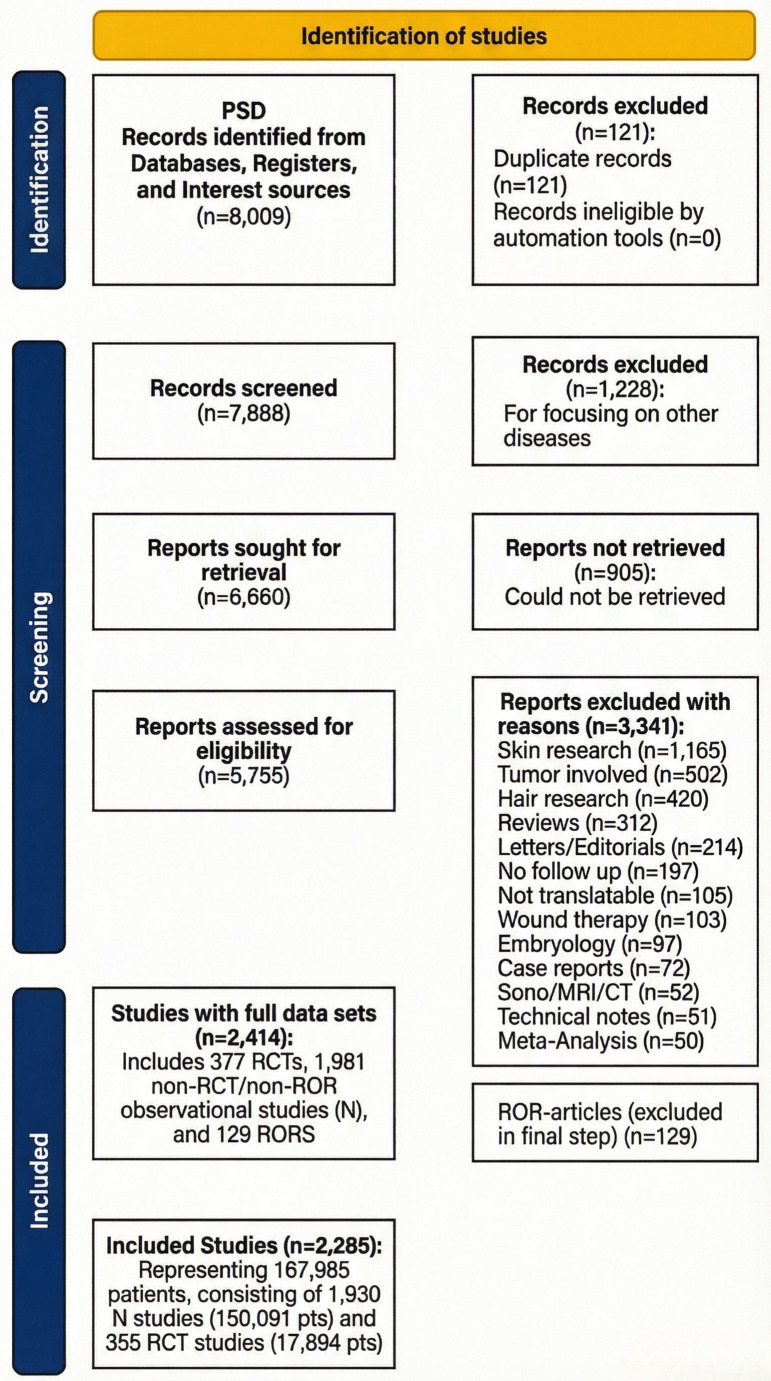
Preferred Reporting Items for Systematic Reviews and Meta-Analysis (PRISMA) flow chart study harvest and qualification steps [6]. PSD: Pilonidal sinus disease, RCT: Randomized controlled trial, ROR: Return on recurrence; GEK`92 = George E. Karydakis’ study 1992 [7]

We included retrospective or prospective clinical series and trials that reported postoperative outcomes in patients undergoing any surgical intervention for PSD and provided a FUP duration metric (mean or median FUP, a defined surveillance window, or sufficient information to derive it). We excluded single-patient case reports, studies limited to non-surgical management, editorials or letters without extractable data, non-human research, and duplicate or overlapping cohorts (retaining the most comprehensive dataset where overlap was suspected). Two reviewers (JS, DD) independently screened titles and abstracts and subsequently evaluated the full texts of potentially eligible reports. Discrepancies were resolved through discussion or adjudication by a third reviewer. Reasons for exclusion at the full-text stage were recorded, and the selection process was summarized in a PRISMA flow diagram (Fig. 1).

### Data extraction and harmonization

Using a piloted extraction form, the bibliographic information (first author, year, journal, country), study characteristics (design, sample size, surgical technique categories), and FUP details (mean and/or median duration, range or interquartile interval, definition of the FUP clock—since surgery versus since wound closure—and methods of outcome ascertainment such as clinic visits, telephone contact, questionnaires, or registry linkage) were recorded. Where recurrence definitions were reported, we recorded whether these were clinical, surgical, or reoperation-based. Any disagreements were reconciled by consensus, and corresponding authors were contacted to clarify ambiguous reporting when necessary. To enable temporal analyses, each study was assigned to a decade using the publication year or mid-decade approximation, as pre-specified. When studies reported FUP since wound closure, we documented this explicitly and considered it in sensitivity analyses; primary analyses emphasized FUP since surgery where available.

### Risk of bias and reporting quality assessment

Because our primary endpoint was a reporting attribute (FUP duration), we appraised methodological quality using tools appropriate to study design with emphasis on FUP adequacy and completeness. For non-randomized studies, we applied the MINORS or JBI case series checklist domains related to FUP, loss-to-FUP, and outcome assessment; for randomized trials, we considered Cochrane RoB 2 domains relevant to attrition and outcome measurement. These appraisals informed sensitivity analyses in which we repeated key estimates after restricting to studies at low or moderate risk.

### Outcomes

The primary outcome was FUP duration expressed in years at the study level. We summarized patient-weighted mean FUP by calendar decade for the period 1970–2020. Secondary outcomes included the proportions of studies and of patients within predefined FUP bands (< 2, 2 to < 5, 5 to < 10, and ≥ 10 years), trends in FUP by study design and surgical technique category, and the association between publication decade and FUP duration.

### Data handling and transformation

We prioritized reported mean FUP for patient-weighted analyses. When only medians were available, we retained them for descriptive summaries and considered established conversion methods in sensitivity analyses, prespecified to avoid introducing systematic bias. For multi-arm studies reporting a single FUP duration, we applied the reported value to each relevant arm while accounting for clustering in weighted analyses using Kish’s effective sample size where appropriate. Suspected cohort overlaps were resolved by retaining the most complete or recent report and excluding duplicates. All time measures were converted to years for consistency.

## Decadal aggregation and patient-weighted means

For each decade, we summarized surveillance using the patient-weighted mean FUP, defined as the ratio of the sum of cohort-size–weighted FUP times to the total number of patients:

$$\overline t(w , d)\;=\;\sum(n_i\cdot\;t_i)\;/\;\sum n_i$$

To evaluate secular trends we applied heteroscedastic weighted least‑squares (WLS):

$$t_i\;=\;\beta_0+\beta_1x_i+\varepsilon_{i\;},\;\;\varepsilon_i\;\sim\;N(0,\;\sigma^2/n_i)$$

Here, x_i_ denotes the calendar year and weights (n_i_) reflect the inverse variance assumption that larger cohorts provide more precise follow‑up estimates. Parameter covariance followed Var(β̂) = σ̂²(XᵀWX)⁻¹ and 95% confidence bands were derived accordingly. All analyses used Python 3.11 (pandas 2.1, statsmodels 0.14), and figures were generated with matplotlib. *N* = 2,074 studies reporting the treatment and FUP of 158,906 patients covered the 1970 decade to today and were further analyzed.

### Statistical analysis

We described FUP duration using means with standard deviations and medians with interquartile ranges. Patient-weighted decadal means were calculated using sample size as the weight, with 95% confidence intervals derived from robust standard errors and verified by nonparametric bootstrap (10,000 resamples) as sensitivity. Temporal trends were estimated using weighted least squares regression of FUP on calendar time (year or decade midpoint), with study sample size as the analytic weight; we reported slope coefficients with 95% confidence intervals and p-values. Unweighted ordinary least squares provided a sensitivity check. We quantified the association between decade and FUP using weighted Pearson correlation, applying Kish’s effective sample size where clustering was present. For the FUP bands, we computed proportions for both studies and patients with binomial confidence intervals and visualized distributions across decades. Prespecified subgroup and sensitivity analyses explored heterogeneity by surgical technique category, study design, geographic region, and risk-of-bias strata. Where multiple hypothesis testing occurred, we controlled the false discovery rate as appropriate. All analyses were conducted in Stata (version 18), and analysis scripts were archived to ensure reproducibility.

## Results

**Fig. 2:**
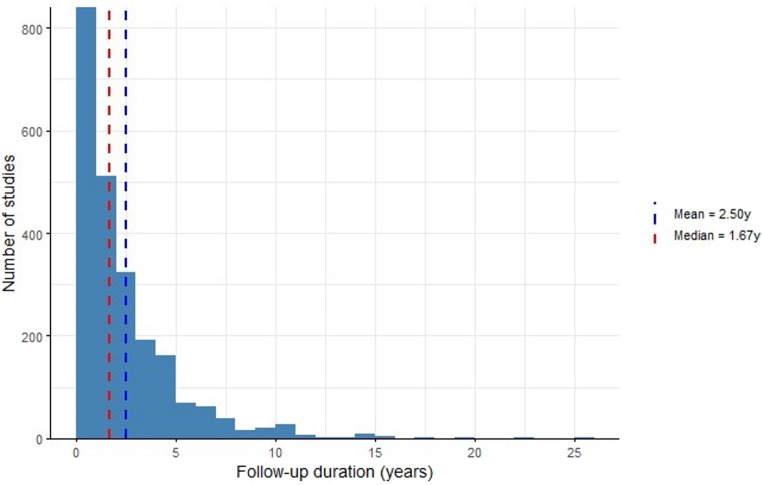
Histogram of reported follow-up times. The red dotted line indicates the median (1.67 years) whilst the blue dotted line shows the mean (2.5 years)

Across 2,285 surgical series spanning 1850–2024, the distribution of reported FUP is markedly right-skewed (Fig. 2). The overall median FUP is 1.67 years, whereas the arithmetic mean is 2.50 years, indicating that a small number of long-surveillance cohorts do not suffice to pull the median above two years. Only 325 of 2,285 studies (14.2%) report ≥ 5 years of observation, and merely 67 (2.9%) achieve ≥ 10 years, underscoring the scarcity of genuinely long-term data. In a stricter count limited to records with complete validation used for trend modeling, 257 studies exceed five years and 53 exceed ten years, corresponding to 11.3% and 2.3%, respectively; these counts are consistent with Fig. 2’s right-skew and with Table 1’s decade summaries.

### Decade-wise patterns and weighted means

**Fig. 3:**
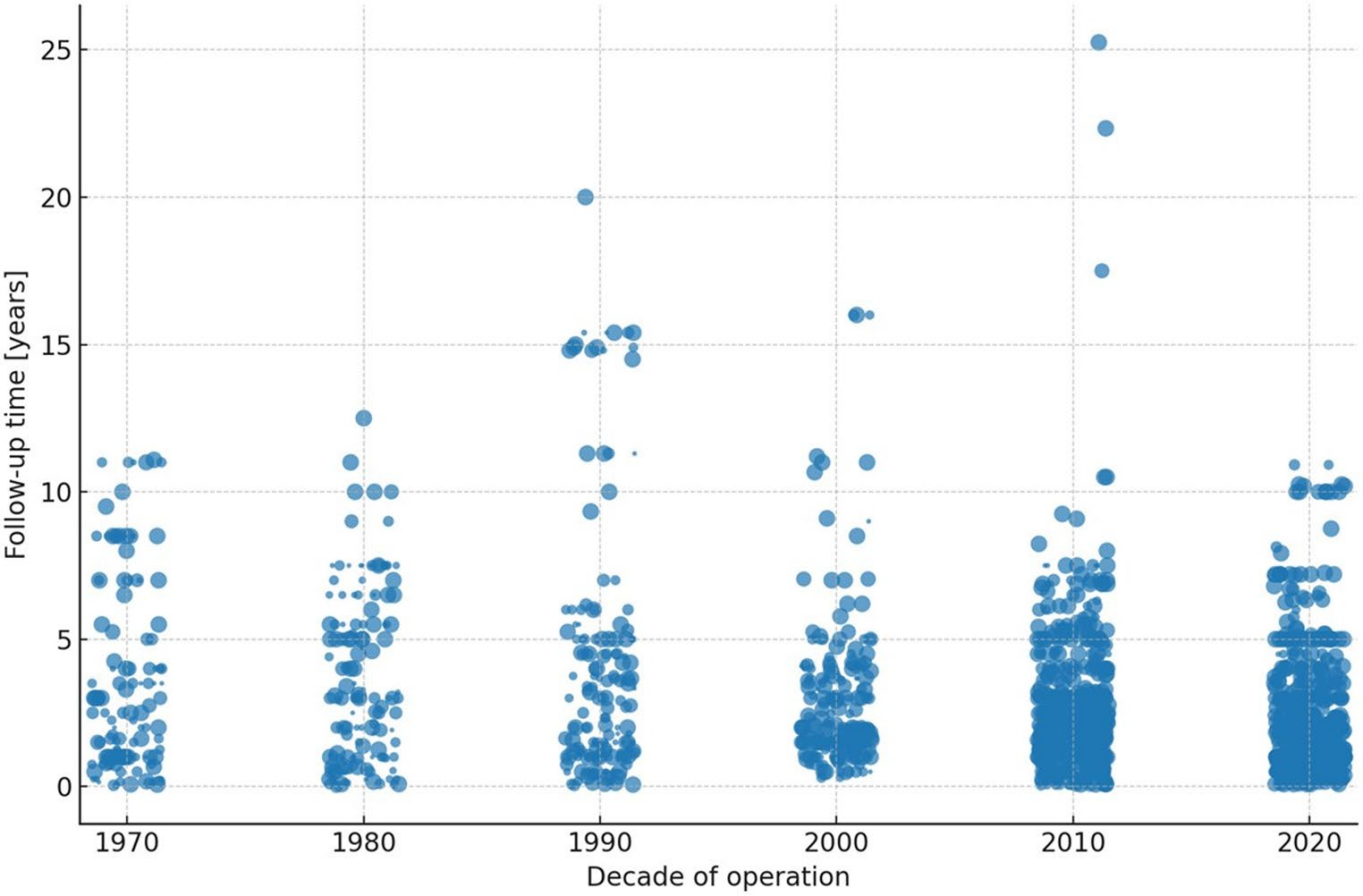
Follow-up time per operative decade since 1970. Points represent studies; with the size equaling the study size. Please note that the 2020 decade is not yet complete

Decade-wise, patient-weighted mean FUP peaks in the 1980s and then declines almost monotonically thereafter (Table 1). Using the patient-weighted estimator we obtain 3.78 years (1970s), 6.69 (1980s), 5.26 (1990s), 4.26 (2000s), 3.14 (2010s), and 2.55 years (2020s) (Fig. 3). Relative to the 1970s, the 2020s are shorter by 1.23 years (− 32.5%); relative to the 1980s peak, the reduction is 4.14 years (− 61.9%).

**Table 1:** Numeric decade‑wise metrics, including total sample size, median, mean and weighted mean FUP (in years)

Decade	Studies	Total N	Median [yrs]	Mean [yrs]	Weighted mean [yrs]
1970	122	9,242	2.38	3.41	3.78
1980	141	13,811	3.00	3.61	6.69
1990	161	9,708	2.67	3.96	5.26
2000	204	15,071	2.00	2.93	4.26
2010	658	57,624	2.00	2.46	3.14
2020	788	53,540	1.13	1.92	2.55

### Secular trend estimates

Figure 3 visualizes the downward shift in study-level FUP from the 1970–1980 s (many cohorts > 4–6 years) to the 2000–2020 s (bulk between ~ 0.5 and 4 years). Larger dots—representing larger cohorts—cluster in recent decades yet are associated with shorter FUP, indicating that contemporary patient volume is concentrated in short-horizon series. The 2010s and 2020s contain the majority of patients (36.3% and 33.6%, respectively), reinforcing the dominance of short follow-up in the modern evidence base.

Formally modeling study-level FUP as a function of calendar year via heteroscedastic weighted least squares (WLS), t_i = β_0 + β_1 x_i + ε_i, with Var(ε_i) = σ² / n_i, yields a weighted slope β_1 = − 0.0766 years per year (95% CI − 0.0856 to − 0.0675; *p* = 4.57 × 10⁻⁵⁸). The unweighted regression gives − 0.0407 years per year (95% CI − 0.0479 to − 0.0334; *p* = 1.47 × 10⁻²⁷). Both models demonstrate a highly significant secular contraction in observed follow-up, with weighing nearly doubling the estimated annual decline—consistent with larger, recent studies having particularly short surveillance. A weighted Pearson correlation between decade and FUP, using Kish effective sample size, is R_w = − 0.342 (n_eff ≈ 216; *p* = 2.5 × 10⁻⁷), corroborating the inverse association (Fig. 3).

### Shares by follow-up band: studies and patients

**Fig. 4:**
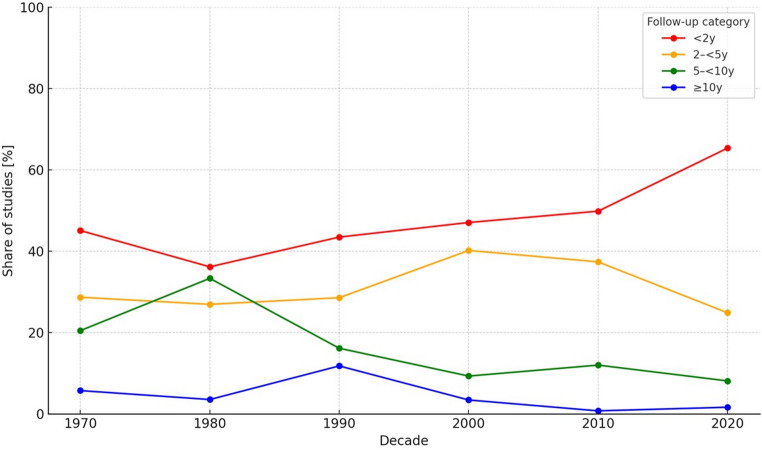
Share of studies by follow-up length, 1970–2020

**Fig. 5:**
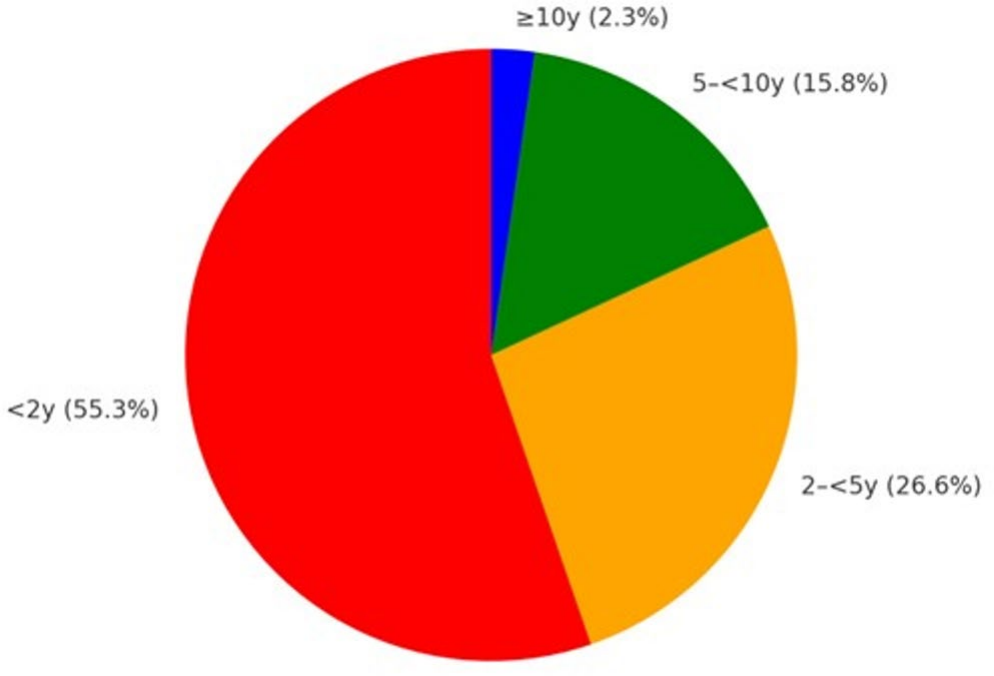
Patient share by follow-up length for 2020 decade

The fraction of studies with < 2 years of FUP rose from 45.1% in the 1970s to 65.4% in the 2020s, while 2–<5 years peaked at 40.2% in the 2000s before settling at 24.9% (Fig. 4). Long-term categories diminished: 5–<10 years declined to 8.1% in the 2020s, and ≥ 10 years is now ~ 1–2% of studies. Patient distribution mirrors these trends (Fig. 5): in the 2020s, 55.3% of patients fall into < 2 years, 26.6% into 2–<5 years, 15.8% into 5–<10 years, and only 2.3% into ≥ 10 years. Thus, the modal experience for contemporary patients is a short observation window, with late recurrences at risk of under-ascertainment.

### Sensitivity and internal consistency

Results are robust to using decade midpoints rather than exact publication years on the x-axis, and to employing n_i as weights versus Kish-adjusted n_eff in the correlation analysis. Across decades, mean values consistently exceed medians, signaling right-skew and occasional long-FUP outliers that do not overturn the central pattern of shortening surveillance. Figure 3’s downward drift is concordant with Table 1’s weighted means and with the WLS slope estimates.

## Discussion

This longitudinal, patient-weighted analysis across 2,285 pilonidal surgery series demonstrates a sustained and statistically robust contraction in postoperative follow-up over the last five decades, with the weighted mean follow-up peaking at 6.69 years in the 1980s and declining to 2.55 years in the 2020s, and with a weighted secular slope of − 0.0766 years per year (95% CI − 0.0856 to − 0.0675; *p* = 4.57 × 10⁻⁵⁸). In parallel, literature has shifted decisively toward short-horizon reporting, with the proportion of studies below two years rising to 65.4% in the 2020s and the share of patients observed for less than two years reaching 55.3%, while truly long-term surveillance beyond ten years has become rare at approximately one to two%. These patterns imply under-ascertainment of late recurrences, which prior cohorts and meta-analyses have shown to accumulate substantially beyond 24 months and up to a decade after closure, rendering early recurrence-free intervals unreliable proxies for durable success [1–3]. Contemporary guideline efforts have recognized this risk and advocate minimum five-year follow-up, strongly encouraging ten-year data whenever feasible, which our findings support and extend by quantifying the secular contraction in patient-weighted terms over successive decades [4, 5].

The paradox that emerges is that publication volume and cohort sizes have expanded markedly since 2000, yet observational horizons have shrunk, concentrating evidence into shorter windows precisely when procedural innovation has accelerated. Several systemic forces likely drive this contraction. Academic acceleration and incentive structures prioritize rapid publication and citation accrual, which disincentivizes sustained surveillance and registry maintenance, and thus favors early reporting of wound healing rather than long-range recurrence capture [8–10]. Increasing patient mobility complicates durable outcome ascertainment and fosters attrition, thereby elevating the risk of misclassification and loss to FUP in the absence of robust tracking mechanisms [11, 12]. The perceived procedural finality of minimally invasive techniques can engender confidence that early closure equates to cure, dampening the motivation to pursue extended observation and potentially shifting outcome emphasis away from decade-long durability [1–3]. Resource and infrastructure limitations also play a role, as long-term registries require coordinated funding and administration in a condition with dispersed care pathways and limited prestige, and this is further challenged by rising incidence and regional variation that call for harmonized surveillance frameworks across health systems [13–15].

Clinically, the downward drift from roughly seven years to about two and a half years of weighted follow-up suggests that a substantial proportion of late failures likely occur beyond the observation window in modern series, especially for techniques introduced after 2000 whose true long-term recurrence profiles are not fully mapped. The narrowing spread of reported follow-up in recent decades should not be misinterpreted as improved homogeneity of outcomes, but rather as convergence toward uniformly short surveillance that reflects accelerated publication cycles and constrained follow-up infrastructure. Methodologically, our use of weighted least squares with study size as the analytic weight appropriately leverages the informational content of larger cohorts, and the agreement in direction and significance between weighted and unweighted models supports the robustness of the central conclusion, even as we acknowledge that weighting presumes precision scales with sample size and that follow-up protocols may vary across studies.

A structural gap that exacerbates these issues is the absence of a PDS COS endorsed by guidelines, which would standardize recurrence definitions, follow-up clocks, ascertainment methods, and minimal reporting milestones. Without a COS, heterogeneity in outcome definitions and follow-up timing hampers comparability and meta-analytic synthesis, undermining decision-grade evaluation across techniques and settings [4–6, 16–19]. Aligning guideline recommendations with a COS set that mandates harmonized two-, five-, and ten-year endpoints, explicit reporting of loss-to-follow-up, and preferred ascertainment pathways including clinical review, telemedicine, and registry linkage would materially improve external validity and ensure that recurrence biology beyond two years is consistently captured.

The implications for clinical practice and research are direct. We recommend mandatory staged reporting at two, five, and ten years for all prospective surgical studies irrespective of randomized or nonrandomized design, along with registry-based multicenter surveillance that minimizes attrition and misclassification through harmonized definitions and periodic audit. Journal and funder policies should explicitly value and incentivize long-term follow-up as a quality metric, counterbalancing existing pressures for rapid output [8–10]. As long-horizon data mature, technique-, age-, and sex-stratified analyses should be prioritized, given the increasing incidence of pilonidal sinus disease, evidence for age-related recurrence effects, and emerging signals of sex-specific vulnerability to recurrence [14, 15, 20–26]. Furthermore, achieving a 10-year follow-up is logistically challenging and often exceeds the standard duration of surgical residencies or fellowship contracts, which inherently limits longitudinal data collection. Additionally, the shift towards minimally invasive techniques may engender a false sense of procedural finality, dampening the motivation to pursue extended observation compared to historical aggressive methods. These measures would recalibrate evidentiary standards toward durability commensurate with the natural history of the disease and provide decision-grade estimates for a predominantly young patient population.

Several limitations warrant consideration. Our analysis is based on study-level summary follow-up rather than individual patient time-to-event data, which precludes formal survival modeling with censoring and limits adjustment for variable loss-to-follow-up. Heterogeneity in recurrence definitions, the choice of follow-up clock—since surgery versus since wound closure—and ascertainment modalities introduces measurement error that likely biases against detecting late events [16–18]. Publication bias cannot be excluded, as series with atypically long or short surveillance may be preferentially published or cited [9, 10]. Weighting by sample size assumes improved precision with larger cohorts, which may not hold where follow-up protocols differ, and estimates for the 2020s remain provisional as the decade is incomplete. While our analysis demonstrates a field-wide systemic contraction in observation windows, follow-up length is heavily influenced by the specific surgical technique utilized. Future prospective studies and long-horizon data must prioritize comparing recurrence patterns and follow-up durations among similar surgical techniques to better isolate method-specific durability. Taken together, these constraints reinforce our central conclusion that the modern literature systematically underestimates true recurrence risk due to contracted observation windows, rather than weakening it.

## Conclusion

In conclusion, median, mean, and patient-weighted follow-up after PDS surgery have decreased substantially since the 1980s, while the share of short-horizon studies and patients has grown, and this contraction risks underestimating true failure rates and overestimating durability for newer techniques. We urge guideline-backed adoption of a COS, mandatory staged reporting at five and ten years, and investment in registry-based, multicenter surveillance to restore decision-grade evidence. Only with durable observation windows consistent with recurrence biology can the surgical community provide robust, evidence-based “good pilonidal practice” care in PDS.

## Data Availability

No datasets were generated or analysed during the current study.

## Abbreviations

PSD: Pilonidal Sinus Disease
FUP: follow up period
COS: core outcome set
